# Danegaptide Prevents TGFβ1-Induced Damage in Human Proximal Tubule Epithelial Cells of the Kidney

**DOI:** 10.3390/ijms22062809

**Published:** 2021-03-10

**Authors:** Paul E. Squires, Gareth W. Price, Ulrik Mouritzen, Joe A. Potter, Bethany M. Williams, Claire E. Hills

**Affiliations:** 1 School of Life Sciences, Joseph Banks Laboratories, University of Lincoln, Lincoln LN6 7DL, UK; PSquires@lincoln.ac.uk (P.E.S.); gprice@lincoln.ac.uk (G.W.P.); jpotter@lincoln.ac.uk (J.A.P.); Bewilliams@lincoln.ac.uk (B.M.W.); 2 Ciana Therapeutics, Ved Hegnet 2, 2960 Rungsted Kyst, Copenhagen, Denmark; umo@cianatx.com

**Keywords:** danegaptide, connexin, hemichannel, ATP, chronic kidney disease, inflammation, fibrosis, hPTECs, TGFβ1

## Abstract

Chronic kidney disease (CKD) is a global health problem associated with a number of comorbidities. Recent evidence implicates increased hemichannel-mediated release of adenosine triphosphate (ATP) in the progression of tubulointerstitial fibrosis, the main underlying pathology of CKD. Here, we evaluate the effect of danegaptide on blocking hemichannel-mediated changes in the expression and function of proteins associated with disease progression in tubular epithelial kidney cells. Primary human proximal tubule epithelial cells (hPTECs) were treated with the beta1 isoform of the pro-fibrotic cytokine transforming growth factor (TGFβ1) ± danegaptide. qRT-PCR and immunoblotting confirmed mRNA and protein expression, whilst a cytokine antibody array assessed the expression/secretion of proinflammatory and profibrotic cytokines. Carboxyfluorescein dye uptake and ATP biosensing measured hemichannel activity and ATP release, whilst transepithelial electrical resistance was used to assess paracellular permeability. Danegaptide negated carboxyfluorescein dye uptake and ATP release and protected against protein changes associated with tubular injury. Blocking Cx43-mediated ATP release was paralleled by partial restoration of the expression of cell cycle inhibitors, adherens and tight junction proteins and decreased paracellular permeability. Furthermore, danegaptide inhibited TGFβ1-induced changes in the expression and secretion of key adipokines, cytokines, chemokines, growth factors and interleukins. The data suggest that as a gap junction modulator and hemichannel blocker, danegaptide has potential in the future treatment of CKD.

## 1. Introduction

Chronic kidney disease (CKD) is a growing health concern associated with increased risk of cardiovascular disease and morbidity [1]. Estimated to affect 10% of the global population [2], risk factors include age, diabetes, hypertension, dyslipidaemia and obesity [3]. The disease is characterised by a decline in the glomerular filtration rate (GFR), in addition to proteinuria, with glomerulosclerosis, tubular atrophy and tubulointerstitial fibrosis (TIF), which are common histopathological changes [4]. Culminating in the loss of epithelial stability, persistent inflammation and increased deposition of the extracellular matrix [5,6], treatment of TIF and advanced CKD represents an unmet clinical need [3]. Consequently, urgent therapeutic approaches are required.

Altered connexin (Cx) expression and function have been implicated in the pathology of various forms of disease (as reviewed in [7]), including CKD (as reviewed in [8,9]). Connexins are a family of membrane-bound proteins that oligomerise into hexameric assemblies termed connexons [10]. When neighbouring cells align, connexons dock to form gap junctions, continuous pores through which messenger molecules can be exchanged, allowing a direct route of communication [10]. When unbound, connexons form hemichannels through which molecules, e.g., adenosine triphosphate (ATP), can be released into the local extracellular space to influence neighbouring cells via activation of purinoreceptors [11]. Expression of the purinergic P2X7 receptor (P2X7R) is upregulated in the renal tubules of individuals with diabetic kidney disease [12] and has been heavily implicated in the progression of fibrosis both in the kidney [13,14,15,16,17] and in other tissue types [18,19,20,21]. Unsurprisingly, targeting purinergic signalling has received considerable attention. However, to date, clinical trials have failed to demonstrate beneficial effects of P2X7R antagonism in numerous inflammatory conditions, an effect perhaps associated with genetic variation within the receptor [22]. Targeting upstream of ATP release would circumvent these problems.

Evidence links the aberrant expression and/or activity of connexins with various forms of CKD [12,23,24,25,26]. Initial studies by Abed et al. reported that heterogenous (Cx43^+/−^) mice, with unilateral ureteral obstruction (UUO), exhibited reduced extracellular matrix (ECM) deposition and decreased inflammation [27], whilst the Cx43-specific mimetic, GAP-26, was able to inhibit monocyte adhesion and blunted the expression of collagen I in the renin transgene (RenTG) mouse model of renin-dependent CKD [25]. More recently, we identified that the predominant connexin isoform expressed in the proximal tubule, Cx43, is elevated in biopsy material isolated from individuals with diabetic nephropathy, an observation paralleled by increased hemichannel-mediated ATP release in TGFβ1-treated primary proximal tubule cells [12]. In evaluating the impact of local increases in extracellular ATP concentrations, we determined that aberrant Cx43-mediated communication initiates disassembly of adherens (e.g., E-cadherin) and tight (e.g., Zona Occludens) junction complexes via activation of P2X7R. These effects, which were negated in a heterogeneous Cx43^+/−^ mouse model of UUO [12], suggest a clear link between Cx43 and tubular injury. Whilst partial ablation of Cx43 confirms a role for connexins in initiating phenotypic changes of tubular injury, pharmacological intervention with the Cx43 hemichannel blocker peptide5 established that the protective nature of this diminished Cx43 activity stems from inhibition of hemichannel-mediated ATP release. The use of mimetic peptides to underscore the protective nature behind blocking hemichannel activity has been successfully reported in other models of disease, including retinopathy [28], neuroinflammation [29], glioma [30,31] and age-related macular degeneration [32,33]. Ultimately, studies link altered connexin activity to increased inflammation [34,35,36] and fibrosis [37,38,39,40] and suggest that stabilising hemichannel- and/or gap-junction-mediated communication (GJIC) may delay the onset of damage observed in various models of injury. Consequently, inhibiting connexin hemichannels provides an attractive means to reduce inflammation and is of considerable therapeutic interest.

Danegaptide ((2S, 4R)-1-(2-aminoacetyl)-4-benzamidopyrrolidine2-carboxylic acid), also known as ZP1609 or GAP-134, is a small dipeptide derived from rotigaptide (AAP10, ZP123). Originally developed as an anti-arrhythmic agent [41], it interacts with Cx43 [42] and acts as a potent gap junction modifier [43]. The compound has demonstrated protective and anti-arrhythmic properties and has previously shown significant effects in established preclinical models of cardiac ischemic reperfusion injuries [44,45]. Whilst phase 2 human clinical trials unfortunately failed to meet their end point [46], studies from other groups have explored the therapeutic potential of danegaptide in other tissue types and models of disease [41,47,48,49]. Moreover, whilst little is known of its role in modulating hemichannel activity, it has been demonstrated that danegaptide reduces hemichannel-mediated dye uptake in C6 glioma cells [43]. Despite this, a role for this compound in blocking hemichannel-mediated ATP release in other tissue types remains to be confirmed.

The current study investigated the efficacy of danegaptide in negating connexin-mediated hemichannel ATP release in primary TGFβ1-treated human proximal tubule epithelial cells (hPTECs), ahead of delineating its protective role against the expression and functional changes commonly associated with progression of tubulointerstitial fibrosis. Using techniques to determine changes in expression (qRT-PCR, immunocytochemistry, immunoblotting, antibody arrays) and function (ATP biosensing, dye uptake, transepithelial electrical resistance, antibody arrays), our findings demonstrate that nanomolar concentrations of danegaptide block Cx hemichannel-mediated ATP release and partly attenuate TGFβ1-induced changes in the expression of cell cycle proteins (e.g., p16, p21, cyclin D1) and reno-protective factors (e.g., Klotho). Moreover, danegaptide decreases the changes often observed in tubular injury, including disassembly of adherens junctions (including a loss of E-cadherin) and tight junctions (including a loss of zona occludens 1 (ZO-1) and claudin 2). Importantly, the proteome profiler array demonstrated the ability of danegaptide to restore changes in the expression and secretion of key extracellular matrix proteins, adipokines, chemokines and growth factors induced by TGFβ1. Consequently, our in vitro data suggest that danegaptide (50–100 nM) can successfully block hemichannel-mediated ATP release in primary hPTECs and partially protect against changes associated with inflammation and fibrosis, as observed in late-stage CKD.

## 2. Results

### 2.1. Danegaptide Does Not Affect Tubular Epithelial Cell Viability

**Figure 1:**
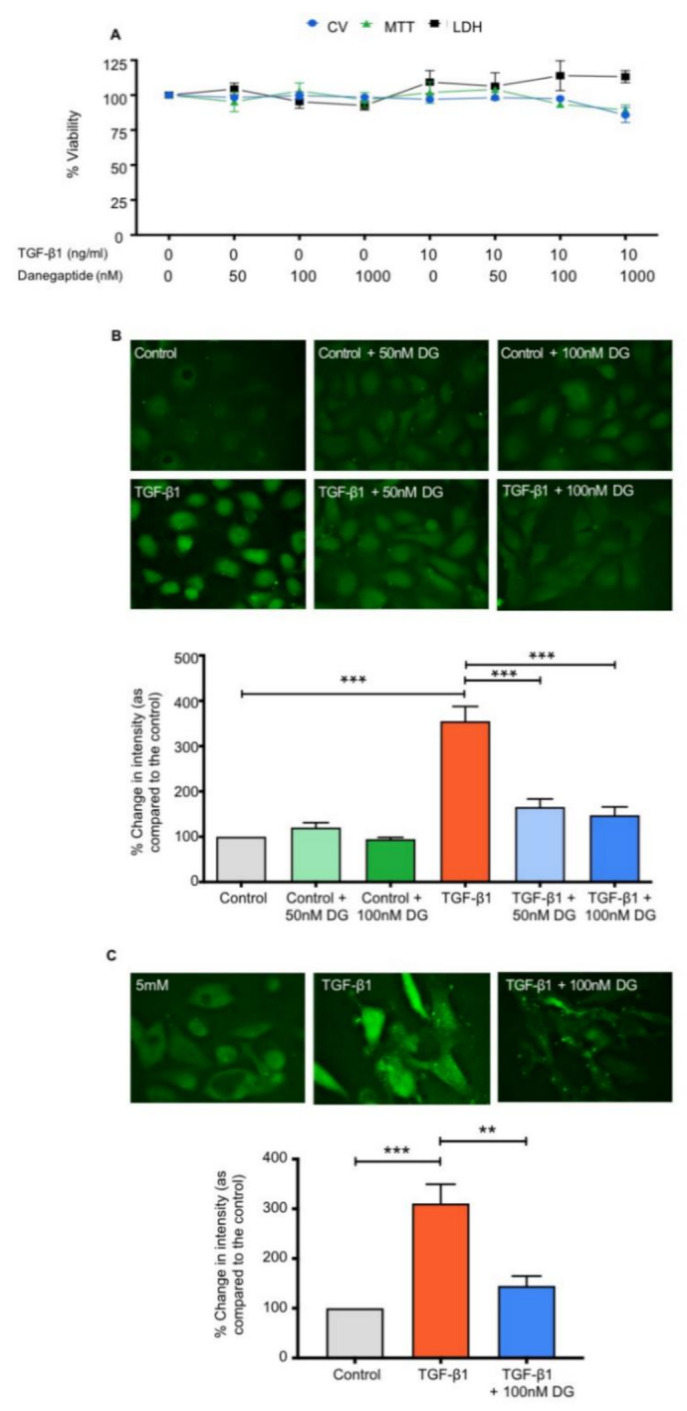
Danegaptide prevents TGFβ1-evoked increases in hemichannel-mediated dye uptake. In panel (**A**), human kidney 2 (HK2) cells were cultured in low glucose (5 mM) ± TGFβ1 (10 ng/mL) ± danegaptide (50, 100 and 1000 nM) for 48 h and cell viability assessed. Results are presented as the mean ± SEM (*n* = 3). Incubation with TGFβ1 (10 ng/mL ± danegaptide (50–1000 nM)) did not alter 3-(4,5-dimethylthiazol-2-yl)-2,5-diphenyltetrazolium bromide (MTT) uptake, lactate dehydrogenase (LDH) release or crystal violet (CV) staining. In panels (**B**) and (**C**), carboxyfluorescein dye uptake was used to assess hemichannel activity in HK2 cells and human proximal tubule epithelial cells (hPTECs), with the degree of dye loading being directly proportional to opening. Cells were cultured in low glucose (5 mM) ± TGFβ1 (10 ng/mL) ± danegaptide (50 or 100 nM) for 48 h. Danegaptide prevented TGFβ1-evoked increases in carboxyfluorescein dye uptake in HK2 cells (panel (**B**)) and hPTECs (panel (**C**)). Minimal dye loading occurred in control cells, whilst dye loading significantly increased in cells treated with TGFβ1. Addition of danegaptide (50 or 100 nM) reduced dye uptake, returning the fluorescence intensity to control levels. Intensity is expressed as a percentage compared to low-glucose controls and is representative of 3 separate experiments. Data are presented as the mean ± SEM (*n* = 3), with key significances indicated (** *p* < 0.01, *** *p* < 0.001; one-way ANOVA and Tukey’s post-test).

Human kidney 2 (HK2) cells were cultured in low glucose (5 mM) for 48 h, prior to being serum-starved overnight and subsequently treated with the optimum concentration of TGFβ1 (10 ng/mL) [12,24] ± danegaptide (50 nM–1 μM) for 48 h (Figure 1A, *n* = 3). A 3-(4,5-dimethylthiazol-2-yl)-2,5-diphenyltetrazolium bromide (MTT) assay confirmed that neither TGFβ1 (101.9 ± 11.7%) nor danegaptide alone altered cell viability (95.2 ± 7% (50 nM), 103 ± 5.7% (100 nM) and 96.6 ± 5.3% (1 μM)) as compared to controls. No effect was observed when TGFβ1-treated cells were co-incubated with danegaptide (104.1 ± 2.2% (50 nM), 93.4 ± 1.6% (100 nM) and 89.3 ± 3.7% (1 μM)). To corroborate these data, a crystal violet (CV) and lactate dehydrogenase (LDH) assay was performed. LDH release in TGFβ1-treated cells was comparable to controls (109.3 ± 11.3%), and co-incubation with danegaptide had no additional effect (106.4 ± 11.6% (50 nM), 113.9 ± 15.6% (100 nM) and 113.2 ± 4.3% (1 μM)). As expected, danegaptide alone did not significantly alter LDH release compared to controls (104.4 ± 4.3% (50 nM), 95.3 ± 4.7% (100 nM) and 92.6 ± 3.3% (1 μM)). Cell staining using crystal violet recapitulated these findings, with data for TGFβ1 (10 ng/mL; 96.9 ± 7.3%) and TGFβ1 plus danegaptide (50 nM–1 μM) being comparable to controls (98.2 ± 1.9% (50 nM), 97.5 ± 1.8% (100 nM) and 85.8 ± 5.3% (1 μM). Lastly, danegaptide alone did not alter crystal violet staining (98.3 ± 2.5% (50 nM), 99.6 ± 2.7% (100 nM) and 98.5 ± 2.2% (1 μM) of controls). In light of these data, a concentration of 50–100 nM was selected for subsequent studies.

### 2.2. Danegaptide Blocks TGFβ1-Evoked Changes in Hemichannel-Mediated Dye Uptake in Tubular Epithelial Cells

We have previously shown that TGFβ1 increases Cx43-mediated hemichannel activity and ATP release from proximal tubule epithelial cells [24]. A carboxyfluorescein dye uptake assay was used to determine whether danegaptide can negate TGFβ1-induced dye uptake through hemichannels in HK2 cells and primary hPTECs. As expected, TGFβ1 (10 ng/mL) increased dye uptake to 354.9 ± 32.6% of controls in HK2 cells, whilst co-incubation with danegaptide (50 nM and 100 nM) significantly blunted the response to 165.5 ± 17.9% and 147.6 ± 18.1%, respectively (Figure 1B; *p* ≤ 0.001, *n* = 4). Danegaptide alone did not alter dye uptake. Carboxyfluorescein dye uptake was increased by TGFβ1 (10 ng/mL) in primary hPTECs (310.8 ± 38.6% of controls), a response partly negated by the co-incubation of danegaptide (100 nM; 145 ± 19.7%) as compared to controls (Figure 1C; *p* ≤ 0.01, *n* = 4).

### 2.3. Danegaptide Negates TGFβ1-Induced Hemichannel-Mediated ATP Release in HK2 Cells

**Figure 2:**
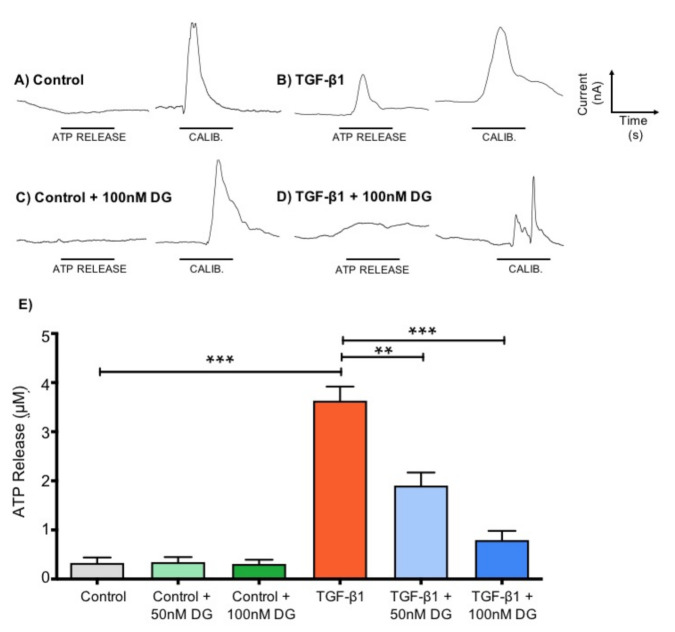
Danegaptide prevents TGFβ1-evoked increases in ATP release. HK2 cells were cultured in low glucose (5 mM) with/without TGFβ1 (10 ng/mL) ± danegaptide (50 or 100 nM) for 48 h. Representative biosensor traces show ATP release following removal of extracellular calcium. Control cells (panel (**A**)) exhibit negligible ATP release compared to a calibration (CALIB) response to 10 μM ATP, whilst a marked increase in release was observed from TGFβ1-treated cells (panel (**B**)). Danegaptide (100 nM) alone failed to alter basal ATP (panel (**C**)) but significantly reduced TGFβ1-evoked ATP release (panel (**D**)). Peak responses were quantified by comparing against a known concentration of ATP (10 µM) and mean data ± SEM plotted (panel (**E**)). Results are representative of 3 separate experiments (*n* = 3; ** *p* < 0.01, *** *p* < 0.001; one-way ANOVA and Tukey’s post-test).

To determine whether danegaptide (50–100 nM) could prevent TGFβ1 (10 ng/mL)-induced release of ATP from hemichannels in HK2 cells, we used ATP biosensing [24,50]. TGFβ1 (10 ng/mL) increased ATP release from 0.33 ± 0.11 µM to 3.60 ± 0.29 µM (Figure 2A,B,E; *p* ≤ 0.001), an effect partially negated by danegaptide at both 50 nM (1.90 ± 0.26 µM; *p* ≤ 0.01) and 100 nM (0.79 ± 0.19 µM; *p* ≤ 0.001) (Figure 2D,E, *n* = 3, six repeats/sample number). Danegaptide alone did not affect ATP release (Figure 2C,E), with ATP levels recorded at 0.35 ± 0.10 µM (50 nM) and 0.31 ± 0.09 µM (100 nM) as compared to controls (Figure 2E).

### 2.4. Danegaptide Reverses TGFβ1-Induced Changes in Cell Cycle Proteins and a Marker of Reno-Protection in hPTECs

**Figure 3:**
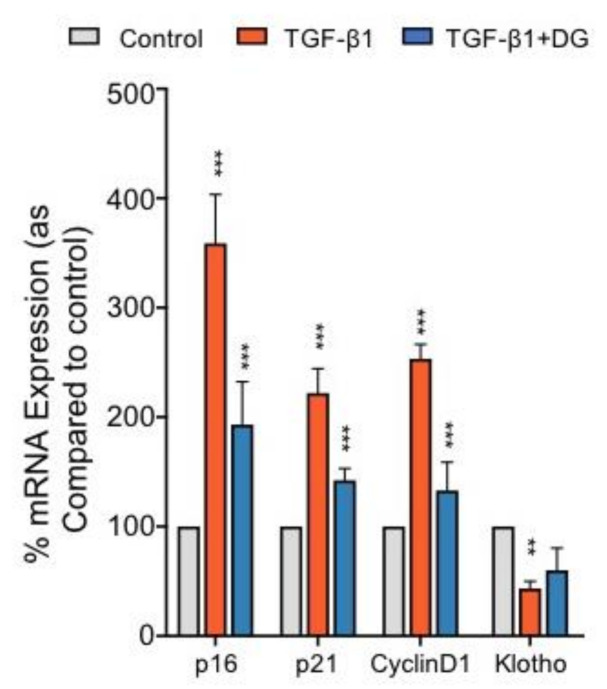
Danegaptide reduces TGFβ1-induced mRNA changes in cell cycle and reno-protective markers. Primary hPTECs were cultured in low glucose (5 mM) ± TGFβ1 (10 ng/mL) ± danegaptide (100 nM) for 12 h. In TGFβ1-treated cells, qPCR analysis demonstrated an increase in p16, p21 and cyclin D1 mRNA and a significant reduction in Klotho mRNA as compared to controls. Danegaptide (100 nM) partly negated the increase in p16, p21 and cyclin D1 and reversed the TGFβ1-evoked change in Klotho. Results are representative of 3 separate experiments and presented as the mean ± SEM (*n* = 3), with key significances indicated (** *p* < 0.01, *** *p* < 0.001; one-way ANOVA and Tukey’s post-test).

To determine whether danegaptide can negate hemichannel-mediated regulation of common cell cycle and reno-protective markers, hPTECs were incubated with TGFβ1 (10 ng/mL) ± danegaptide (100 nM) for 12 h and the expression of candidate gene mRNA assessed through qPCR analysis. Addition of danegaptide to TGFβ1-treated hPTECs returned the expression of p16 from 358.8 ± 21.1% to 193 ± 18.5% of controls, p21 expression from 221.8 ± 12.9% to 142.2 ± 6.2% and cyclin D1 expression from 253.2 ± 7.7% to 132.9 ± 15.0% (Figure 3; *p ≤* 0.001, *n* = 3). In addition, danegaptide partially reversed the decline in Klotho from 43.3 ± 3.8% to 59.9 ± 11.7% of controls (Figure 3, *n* = 3).

### 2.5. Danegaptide Restores TGFβ1-Mediated Changes in Adherens and Tight Junction Proteins and Paracellular Permeability in hPTECs

**Figure 4:**
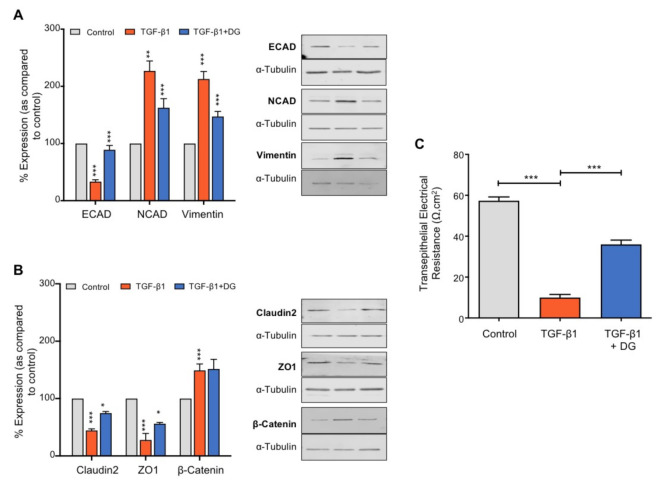
Danegaptide reduces TGFβ1-evoked changes in adherens and tight junction proteins and epithelial leakiness. Primary hPTECs were cultured in low glucose (5 mM) ± TGFβ1 (10 ng/mL) ± danegaptide (100 nM) for 48 h. Expression of E-cadherin (ECAD), N-cadherin (NCAD) and vimentin (panel (**A**)) and of claudin-2, zona occludens 1 (ZO-1) and β-catenin (panel (**B**)) was assessed via Western blotting. TGFβ1 reduced E-cadherin, claudin-2 and ZO-1 expression and increased N-cadherin and vimentin expression. Effects were partially reversed by danegaptide (100 nM). Representative blots for each protein are shown, with expression normalised by re-probing for ɑ-tubulin as a loading control. Results are presented as the mean ± SEM (*n* = 3), with key significances indicated (* *p* < 0.05, ** *p* < 0.01, *** *p* < 0.001; one-way ANOVA and Tukey’s post-test). In panel (**C**), transepithelial electrical resistance (TER) assessed the consequence of altered adherens and tight junction protein expression on epithelial integrity. HK2 cells were cultured in low glucose (5 mM) on Transwell inserts and transepithelial resistance measured. TGFβ1 reduced TER, an effect partially restored by the addition of danegaptide (100 nM). Data are expressed as the mean ± SEM (*n* = 3; *** *p* < 0.001).

In the kidney, reduced E-cadherin (ECAD)-mediated cell adhesion initiates a series of events that culminate in an intermittent phenotype associated with partial epithelial-to-mesenchymal transformation. Initiation facilitates disassembly of both adherens and tight junction complexes, culminating in loss of adhesion, diminished gap junction intercellular communication and leaky epithelia [12,24,51]. To determine whether danegaptide can negate TGFβ1-mediated changes in adherens and tight junction proteins, hPTECs were incubated with TGFβ1 (10 ng/mL) ± danegaptide (100 nM) for 48 h and expression of candidate proteins assessed. Danegaptide partially restored E-cadherin expression from 33.3 ± 3.3% to 89 ± 7.6% of controls, N-cadherin (NCAD) expression from 224.4 ± 29.6 to 161.9 ± 27.4% and vimentin expression from 212.9 ± 13% to 147.3 ± 8.8% (Figure 4A; *p* ≤ 0.001, *p* ≤ 0.01 and *p* ≤ 0.001, respectively; *n* = 3). β-catenin expression remained unaltered by the hemichannel blocker from 149.2 ± 11.2% (TGFβ1 alone) to 151.6 ± 16.8% (TGFβ1 + danegaptide, Figure 4B; *p* = *NS, n* = 3). Assessment of the effects of danegaptide on tight junction proteins confirmed that the gap junction modulator partially restored the expression of claudin-2 from 44.5 ± 5% to 74.6 ± 5% of controls and ZO-1 from 27.8 ± 11.3% to 52.8 ± 5.4% as compared to controls (Figure 4B; *p* ≤ 0.05, *n* = 3). Studies examining transepithelial electrical resistance confirmed that reduced expression of tight junction proteins seen with TGFβ1 (10 ng/mL) is paralleled by a loss of transepithelial resistance from 57.33 ± 1.86 Ω·cm^2^ to 10 ± 1.53 Ω·cm^2^ (*p* ≤ 0.001, *n* = 3). This increased leakiness was partially corrected by co-incubation with danegaptide (36 ± 2.08 Ω·cm^2^) (Figure 4C; *p* ≤ 0.001, *n* = 3).

### 2.6. Danegaptide Prevents TGFβ1-Evoked Upregulation of Extracellular Matrix Proteins in hPTECs

**Figure 5:**
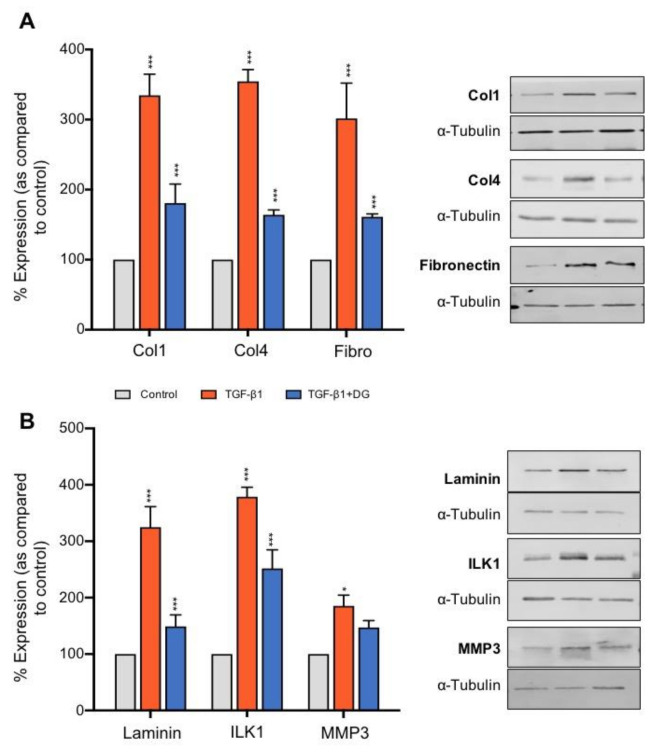
Danegaptide negates TGFβ1-evoked changes in the expression of proteins associated with the extracellular matrix. Primary hPTECs were cultured in low glucose (5 mM) ± TGFβ1 (10 ng/mL) ± danegaptide (100 nM) for 48 h. In panel (**A**), expression of collagen I (Col1), collagen IV (Col4), fibronectin (Fibro) and laminin (panel (**B**)) was assessed via Western blotting. TGFβ1 upregulated extracellular matrix (ECM) protein expression, an effect reduced by danegaptide (100 nM). Similarly, danegaptide partially reversed TGFβ1-evoked changes in matrix metalloproteinase 3 (MMP3) and integrin-linked kinase 1 (ILK1). Representative blots are shown, with expression normalised by re-probing for ɑ-tubulin as a loading control. Bars correspond to their associated lanes in the respective blot. Results are presented as the mean ± SEM (*n* = 3; * *p* < 0.05, *** *p* < 0.001; one-way ANOVA and Tukey’s post-test).

Caused by an imbalance between formation and degradation, the accumulation of the ECM is a major hallmark of CKD. A role for TGFβ1 in this process is well established [52], and understanding how to negate these changes has clear implications to progression of the disease. To examine the effect of danegaptide on TGFβ1-evoked changes in the expression of ECM proteins, hPTECs were cultured in low glucose (5 mM) for 48 h, serum-starved overnight and treated with TGFβ1 (10 ng/mL) ± danegaptide (100 nM) for 48 h. Compared to controls, TGFβ1 increased the expression of the ECM proteins collagen I (334.6 ± 30.14%), collagen IV (354.5 ± 16.9%), fibronectin (301.7 ± 50.4%) and laminin (324.8 ± 36.4%) (Figure 5A,B; *p* ≤ 0.001, *n* = 3). Co-incubation with danegaptide significantly attenuated these changes, restoring the expression to 180.7 ± 27.3% (collagen I), 164 ± 6.9% (collagen IV), 161.3 ± 4% (fibronectin) and 149 ± 20.4% (laminin) (*p* ≤ 0.001; *n* = 3 in each case). Danegaptide (100 nM) also reduced TGFβ1 (10 ng/mL)-evoked changes in integrin-linked kinase 1 (ILK1) from 378.9 ± 16.8% to 251.8 ± 33% (*p* ≤ 0.001) but had minimal effect on matrix metalloproteinase 3 (MMP3), reducing the expression from 185.3 ± 19.6% with the cytokine alone to 147.1 ± 12.2% when co-incubated with danegaptide (Figure 5B).

### 2.7. Danegaptide Reduces TGFβ1-Evoked Changes in the Expression of Adipokines, Chemokines, Growth Factors and Interleukins from hPTECs

**Figure 6:**
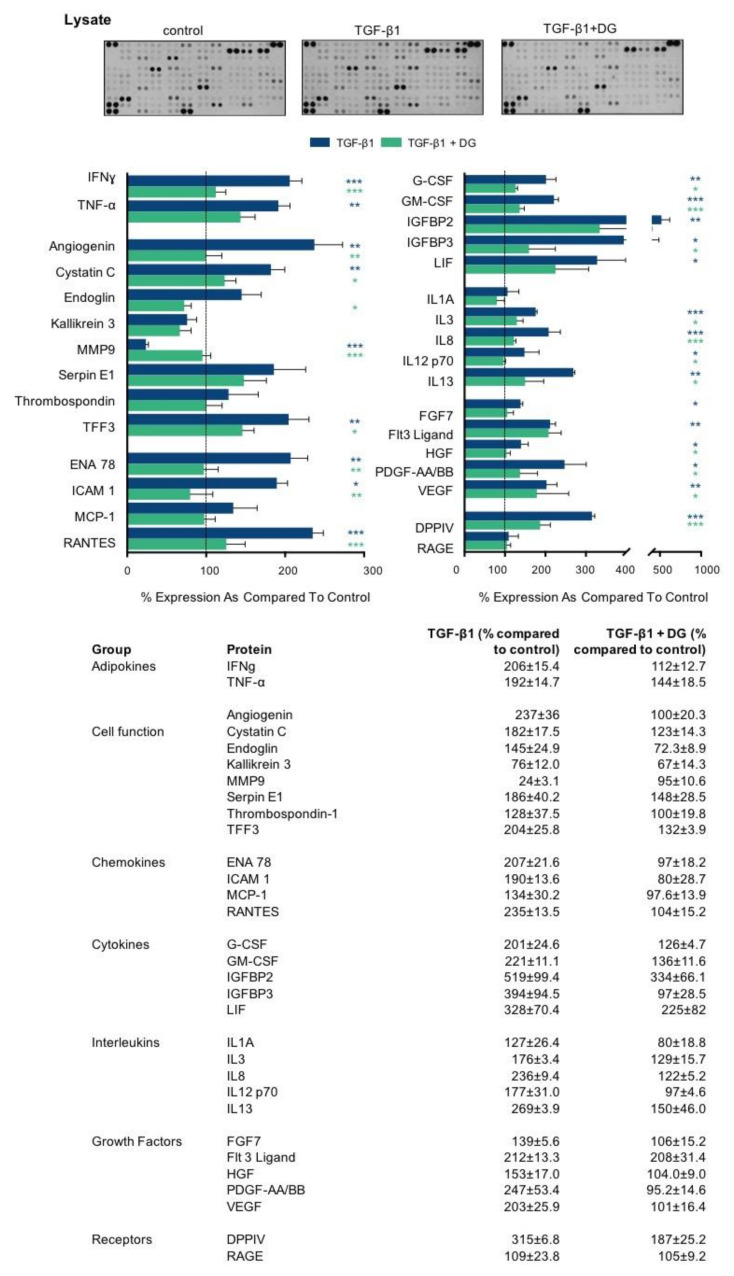
Danegaptide prevents TGFβ1-evoked changes in the expression of adipokines, chemokines, growth factors and interleukins. Primary hPTECs were cultured in low glucose (5 mM) ± TGFβ1 (10 ng/mL) ± danegaptide (100 nM) for 48 h. An inflammation antibody array was used to assess the expression of 31 candidate inflammatory proteins in hPTEC lysates. Results are representative of 3 separate experiments and presented as the mean ± SEM (*n* = 3), with key significances indicated (* *p* < 0.05, ** *p* < 0.01, *** *p* < 0.001; one-way ANOVA and Tukey’s post-test).

**Figure 7:**
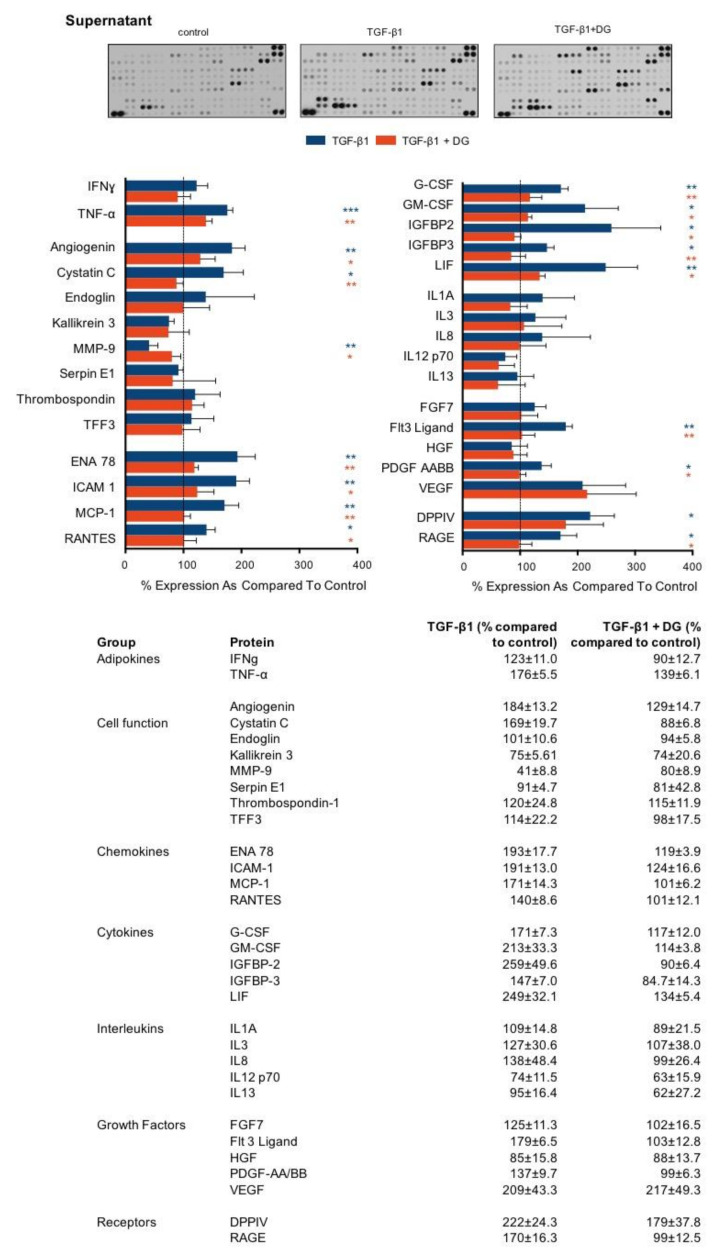
Danegaptide prevents TGFβ1-evoked changes in the secretion of adipokines, chemokines, growth factors and interleukins. Primary hPTECs were cultured in low glucose (5 mM) ± TGFβ1 (10 ng/mL) ± danegaptide (100 nM) for 48 h. An inflammation antibody array was used to assess the secretion of 31 candidate inflammatory proteins in the supernatant from treated hPTECs. Results are representative of 3 separate experiments and presented as the mean ± SEM (*n* = 3), with key significances indicated (* *p* < 0.05, ** *p* < 0.01, *** *p* < 0.001; one-way ANOVA and Tukey’s post-test).

The inflammatory response in and around proximal tubules involves both the activation of multiple cell types and the secretion of numerous inflammatory markers. Specifically, soluble chemokines, cytokines and growth factors recruit and activate infiltrating immune cells and stimulate resident fibroblasts. Sustained activation of these cells mediates tubulointerstitial fibrosis. We used the proteome profiler array to determine whether danegaptide negates TGFβ1-induced changes in the expression and secretion of key proinflammatory mediators. Primary hPTECs were cultured, as described above, and treated with TGFβ1 (10 ng/mL) ± danegaptide (100 nM) for 48 h. A list of changes in lysate (Figure 6) and supernatant (Figure 7) are provided for 31 candidate proteins grouped by primary function.

## 3. Discussion

In 2017, the global incidence of CKD was at 697.5 million cases, with the condition identified as the 12th-leading cause of death worldwide [53]. To date, there are no curative therapeutic options for end-stage renal disease (ESRD). Furthermore, with low dialysis survival rates and a global shortage of kidney donors, there is a pressing need to develop therapies that could prevent CKD progression and improve the patient’s quality of life.

In CKD, the severity of inflammation in the proximal tubule dictates how quickly a kidney will fail. Contributed to by the activation of multiple cell types, it appears, in part, to involve connexin-mediated cell-to-cell communication [9]. Connexins facilitate direct and local paracrine-mediated cell communication through their ability to oligomerise into hexameric connexons. When neighbouring cells align, connexons dock to form gap junctions [10]. These continuous channels provide a direct route for information transfer, allowing cells to lock into a particular frequency and synchronise activity. In contrast to gap junctions, which typically open under physiological conditions, undocked connexons, also termed hemichannels, have a low open probability and open in response to injury [10]. Altered hemichannel activity is associated with the pathophysiology of multiple disease states, with evidence linking altered cell-to-cell communication to increased senescence [54], inflammation [34,55,56] and fibrosis [18,19,20,21]. Data from our own lab demonstrate that the predominant connexin isoform in the proximal tubule (Cx43) is up-regulated in advanced CKD and correlates with elevated levels of TGFβ1 and severity of fibrosis and inflammation [24]. Moreover, this altered expression translates to a loss of gap junction intercellular communication, which is accompanied by increased hemichannel-mediated ATP release and changes indicative of early tubular injury. Consequently, blocking Cx43-mediated hemichannel ATP release may represent a viable target for treatment of the late-stage damage that develops in individuals with CKD.

Danegaptide is a small therapeutic peptide that until recently has been used in its capacity to restore gap junction coupling in multiple models of injury, including ischaemia [43,44] and retinopathy [49]. Danegaptide has been shown to block hemichannel activity and dye uptake in C6 glioma cells [43]; however, a role for the compound in blocking hemichannel-mediated ATP release remains to be confirmed, and no studies have yet evaluated the effects of danegaptide on injured kidney tubules. In the current study, we presented compelling evidence that danegaptide blocks TGFβ1-induced hemichannel-mediated ATP release in primary human proximal tubule epithelial cells. Furthermore, the compound restored TGFβ1-evoked changes in the expression and secretion of proteins linked to inflammation and fibrosis. In CKD, tubulointerstitial fibrosis develops in response to various morphological and phenotypic changes, including epithelial-to-mesenchymal transition (EMT), inflammatory cell infiltration, fibroblast activation and extracellular matrix (ECM) remodelling [57]. Recent evidence suggests that cellular senescence may play a key role in the progression of chronic kidney disease [58], with senescence linked to EMT, a proinflammatory secretome, and extracellular matrix deposition [59,60,61,62]. Senescence denotes irreversible proliferative growth arrest with associated changes in chromatin organisation, gene transcription and protein secretion [63,64]. Consequently, senescent cells are known to exhibit increased expression of cyclin-dependent kinase (CDK) inhibitors (CKIs), including p21^Cip1^ (p21) and p16^Ink4a^ (p16), and altered expression of reno-protective Klotho [65,66,67,68]. In the current study, mRNA expression of CDK inhibitors p16, p21 and cyclin D1 was significantly elevated in TGFβ1-treated tubular epithelial cells, whilst the reno-protective anti-aging protein Klotho demonstrated reduced expression compared to controls. Interestingly, the expression of p16, p21 and cyclin D1 was ameliorated when cells were co-incubated with danegaptide. The importance of this observation is supported by numerous in vivo studies [69,70], including recent work in the *p16 ^INK4a^* double-knockout mouse model. When induced with UUO to exhibit advanced interstitial inflammation and fibrosis, these mice exhibited decreased apoptosis, senescence, diminished levels of TGFβ1/Smad signalling and a reduction in inflammatory cell infiltration as compared to wild-type animals [70]. Moreover, proximal tubule cells isolated from the UUO model exhibited increased levels of p21, suppression of which is paralleled by a reduction in markers commonly associated with EMT [71,72]. A direct link to cell senescence and induction of EMT has already been established [73].

The loss of E-cadherin-mediated cell adhesion is an initiating trigger of EMT, with a concomitant increase in N-cadherin (the cadherin switch), accompanied by disassembly of the adherens junction and acquisition of proteins commonly associated with a fibroblast phenotype, e.g., vimentin and fibroblast-specific protein [74]. In the kidney, it is well established that tubular injury evokes a number of morphological and phenotypic changes characteristic of partial, if not full, EMT. Our recent studies demonstrated that glucose-evoked changes in TGFβ1 mediate disassembly of the adherens and tight junction complex by regulating changes in adherens (ECAD, NCAD, β-catenin) and tight junction (ZO-1) protein expression. Moreover, in vitro administration of non-hydrolysable ATP downregulated E-cadherin expression in proximal kidney cells, the loss of which was paralleled by a reduction in intercellular ligation forces, decreased tether rupture events and cytoskeletal remodelling [51]. Mediated by P2X7R, the effects were reversed in the heterogenous Cx43^+/-^ UUO model [12]. Building on these findings, the effectiveness of danegaptide underscores a protective role for blocking hemichannel-mediated ATP release in preventing altered expression of key epithelial (ECAD, β-catenin, ZO-1, claudin 2) and mesenchymal (NCAD, vimentin) proteins, the implications of which are of therapeutic interest.

The adherens junction assembles when the cytoplasmic tail of E-cadherin binds to β-catenin, mediating attachment to the cytoskeletal network and ensuring that cell polarity and architecture are maintained [75]. Despite its role in maintaining cell polarity, disassembly of these junctions allows for the release of β-catenin into the cytosol, which when activated by either Wnt proteins or other upstream regulators, e.g., integrin-linked kinase (ILK), can translocate into the nucleus and regulate cell-specific effects. Integrin-linked kinase is an intracellular serine/threonine protein kinase that plays a fundamental role in the regulation of cell adhesion, survival, proliferation and extracellular matrix (ECM) deposition [76]. Importantly, inhibition of ILK attenuates renal fibrosis in multiple models of CKD [77]. In the current study, TGFβ1-induced increases in ILK1 expression were, in part, restored when cells were co-incubated with danegaptide, an effect that paralleled a decrease in β-catenin expression and ECM-related proteins. Interestingly, whilst danegaptide failed to significantly alter the TGFβ1-induced increase in β-catenin expression, it is important to note that the absence of a change in expression does not reflect the absence of its function within the cell. The canonical Wnt pathway involves the nuclear translocation of β-catenin and activation of target genes via a group of transcription factors called the T cell factor/lymphoid enhancer factors (TCF/LEF) [78]. Normally, Wnt/β-catenin signalling is silent, but it is reactivated after injury in a number of different models of chronic kidney disease [79,80]. Moreover, aberrant Wnt/β-catenin signalling is highly associated with the initiation of senescence, an effect that parallels the loss of expression of Klotho, an anti-aging protein that acts as a negative regulator of the canonical Wnt pathway [81], and in the current study was downregulated in TGFβ1-treated cells (Figure 3). Consequently, sustained activation of Wnt/β-catenin signalling is linked to the progression of fibrosis in both the kidney and other tissue types [79,82,83]. Once translocated into the nucleus, β-catenin is associated with increased expression of the transcription factor SNAIL and the matrix metalloproteinase MMP7. Interestingly, these events are associated with transcriptional repression and extracellular domain membrane shedding of E-cadherin, the latter of which sees an increase in the cytoplasmic pool of free β-catenin [84]. Accordingly, Wnt signalling is a key activator of EMT in conditions of injury [85]. Moreover, with evidence that Wnt/β-catenin signalling promotes the expression of numerous genes, including fibronectin, FSP1, Snail1, MMP7 and cyclin D1, it is not surprising that attenuation of this signalling cascade is associated with improved renal function, reduced EMT [86] and diminished inflammation [87] and fibrosis [80,88]. Consequently, it could, in part, account for the TGFβ1-induced increased expression and secretion of extracellular matrix proteins (Figure 5), proinflammatory cytokines and chemokines observed in this study (Figure 6 and Figure 7). Increased ECM deposition and secretion of proinflammatory molecules are hallmarks of tubulointerstitial fibrosis, with different cell types, including renal tubular epithelial cells known to secrete a large number of factors that are collectively defined as the CKD-associated secretory phenotype (CASP). Characterised by numerous molecules, including interleukins, extracellular matrix proteins and chemokines, this CASP bares striking similarities to the senescence-associated secretory phenotype (SASP), with components of the CASP having been strongly associated with the pathology of tubulointerstitial fibrosis [89]. When released, these proinflammatory factors act upon neighbouring healthy cells in a paracrine fashion, thereby driving the progression of fibrosis in CKD. As many factors associated with the CASP are known to induce fibrosis in the kidney (e.g., TGFβ, interleukin (IL)-1, and interleukin (IL)-6), targeting upstream of these inflammatory signalling intermediates might prove an effective alternative strategy for CKD treatment. Using a proteome profile array, the cell supernatant from TGFβ1±danegaptide-treated cells was incubated on a cell membrane preabsorbed with antibodies raised against key inflammatory proteins. As highlighted in Figure 6 and Figure 7, co-incubation of cells with TGFβ1 and danegaptide restored the expression and secretion of a number of candidate proteins whose role in kidney disease is well established as either detrimental (tumour necrosis factor-alpha, interleukin 1-alpha, interferon-gamma) or protective (hepatocyte growth factor). Moreover, in CKD, soluble chemokines, adhesion molecules and growth factors recruit and activate infiltrating immune cells and resident fibroblasts to mediate inflammation and fibrosis in the injured kidney. Our array data confirm that modulating Cx43 and blocking hemichannel-mediated ATP release in tubular epithelial cells negates, either in part or fully, secretion of many inflammatory mediators, including chemokines, monocyte chemo-attractant protein (MCP1) and Regulated on Activation, Normal T Cell Expressed and Secreted (RANTES), both of which are involved in monocyte recruitment. Elevated in both human and experimental kidney diseases, MCP1 secretion is triggered by interleukin-1, tumour necrosis factor-alpha [90] and interferon-gamma [91], all of which were induced in our model by TGFβ1, yet blocked when co-applied with danegaptide. Moreover, danegaptide was also observed to prevent TGFβ1-induced secretion of intercellular cell adhesion molecule (ICAM1) [92], granulocyte-macrophage colony-stimulating factor (GM-CSF) [93] and epithelial-neutrophil-activating peptide (ENA78) [94]. Collectively, these have been linked to the progression of CKD and with other key pathogenic proteins, including dipeptidyl peptidase 4 (DPPIV) [95] and vascular endothelial growth factor (VEGF) [96], and have been identified as potential therapeutic targets. Despite these observations, array analysis identified a number of proteins at 48 h, where TGFβ1-induced regulation appeared to independent of hemichannel-mediated ATP release. As evidenced by the expression of the FLT3 ligand in the cell lysate, danegaptide failed to significantly alter TGFβ1-induced changes in whole-cell expression. The FLT3 ligand is initially synthesised as a membrane-bound protein, which must be cleaved to become a soluble growth factor. In TGFβ1-treated cells, an increase in membrane-bound FLT3L was observed compared to controls in the cell lysate, a response that was unaffected when cells were co-incubated with danegaptide. Although little is known about the enzyme involved in the proteolytic cleavage and release of FLT3L, a study by Horiuchi et al. demonstrated that shedding of FLT3L and release from the membrane are metalloprotease dependent and that this effect in fibroblasts is dependent on the TNFα-converting enzyme (TACE) [97]. Whilst we can only speculate, it is possible that blocking Cx43-mediated ATP release may blunt the activity of a protein(s) required for FLT3 membrane shedding and, thus in the absence of FLT3L release, FLT3L levels in the supernatant are significantly less in TGFβ1+danegaptide-treated cells as compared to TGFβ1-treated controls. Interestingly, whilst danegaptide did not appear to reverse the TGFβ1-induced increase in the FLT3L cell lysate, it did have an effect on the secreted form of the protein.

The soluble Receptor for Advanced Glycation Endproducts (RAGE) is a potential biomarker of inflammation and oxidative stress [98]. It acts as a decoy receptor that prevents advanced glycation end products binding to membrane-bound RAGE and RAGE-related detrimental effects. In the current study, we observed a 70% increase in the secretion of sRAGE with TGFβ1, an effect restored to control by danegaptide. sRAGE is functional and able to induce an effect when secreted. The potential of danegaptide to significantly negate this secretion is of clear therapeutic interest. Interestingly, however, we did not observe an increase in the RAGE lysate at 48 h with TGFβ1 ± danegaptide. Whilst these experiments have been performed in human primary proximal tubule cells, the limitations of our model may have cell-, time- and concentration-dependent effects. Consequently, whilst a better understanding of the mechanism of action of danegaptide is needed, findings from this study provide important initial evidence of the benefits of using danegaptide to negate TGFβ1-induced changes in markers of inflammation and tubular injury via blockade of hemichannel-mediated ATP release. We concede that our in vitro data provide a minimalistic model of the multifactorial events that give rise to tubulointerstitial fibrosis, and recommend caution in translating these novel findings to the in vivo situation, especially where studies on other species and models of injury have demonstrated variable effects [41,44,46,47,48,49]. Further studies are now required to determine the selectivity of these hemichannels and the capability of the drug in preclinical models of chronic damage, ahead of testing efficacy in human clinical trials.

## 4. Materials and Methods

### 4.1. Materials

Clonal human kidney 2 (HK2) epithelial cells and primary human proximal tubule epithelial cells (hPTECs) were purchased from the American Type Culture Collection (ATCC) (LGC Standards). Tissue culture supplies were purchased from Invitrogen (Paisley, UK). The Immobilon-FL PVDF membrane was from Millipore (Watford, UK), and Odyssey blocking buffer and secondary fluorescent antibodies were purchased from LI-COR (Cambridge, UK). Antibodies for E-cadherin, N-cadherin, ILK1, MMP-3, β-catenin, vimentin and ZO-1 were obtained from Cell Signalling Technologies (Hertfordshire, UK), whilst claudin-2, laminin, collagen I and collagen IV antibodies were obtained from ABCAM (Cambridge, UK). Fibronectin antibody was purchased from Santa Cruz (Santa Cruz, CA, USA).

Recombinant hTGFβ1 was obtained from Sigma (Poole, UK), as were all other general chemicals. Danegaptide was provided by Zealand Pharmaceuticals. ATP biosensors were from Sarissa Biomedical Ltd. (Coventry, UK) and fluorodishes from WPI (Hertfordshire, UK). Transwell filters were purchased from Corning (Nottinghamshire, UK). The Proteome Profiler Human Cytokine Array Kit was from R&D Systems (Oxfordshire, UK).

### 4.2. Tissue Culture

Primary human proximal tubule epithelial cells (hPTECs) were maintained in a renal epithelial cell basal medium from the ATCC, supplemented with 0.5% fetal calf serum (FCS wt/vol, triiodothyronine (10 nM), rhEGF (10 ng/mL), hydrocortisone hemisuccinate (100 ng/mL), rhInsulin (5 μg/mL), epinephrine (1 μM), transferrin (5 μg/mL) and L-alanyl-L-glutamine (2.4 mM), in a humidified atmosphere at 37 °C with 5% CO_2_. Cells were subjected to overnight serum starvation prior to treatment. Human kidney 2 (HK2) cells (passages 18–30) were grown in Dulbecco’s Modified Eagle’s Medium (DMEM)/Hams F12 medium, supplemented with 10% FCS wt/vol, glutamine (2 mmol/L) and epithelial growth factor (EGF) (5 ng/mL), in a humidified atmosphere at 37 °C with 5% CO_2_. HK2 cells were immortalised by the transduction of human papilloma virus 16 (HPV-16) E6/E7 genes and were mycoplasma free. In all experiments, cells were seeded in low-glucose DMEM/F12 (5 mmol/L) for 48 h and then serum-starved overnight prior to treatment.

### 4.3. MTT Assay

The 3-(4,5-dimethylthiazol-2-yl)-2,5-diphenyltetrazolium bromide (MTT) assay was performed, as described previously, to assess the cytotoxic effects of danegaptide on cell proliferation [99]. HK2 cells were seeded in 96-well plates and cultured in low-glucose DMEM/F12 (5 mM) for 48 h, prior to overnight serum starvation, and then subsequently stimulated for 48 h with TGFβ1 (10 ng/mL) ± danegaptide (50–1000 nM). Colourimetric measurement of formazan production corresponded to the number of viable cells.

### 4.4. Lactate Dehydrogenase Assay

The release of lactate dehydrogenase (LDH) into media as a result of plasma membrane damage is commonly used to evaluate cell death or cytotoxicity. HK2 cells were seeded in 96-well plates and cultured in low-glucose DMEM/F12 (5 mM) for 48 h prior to overnight serum starvation. Cells were then stimulated for 48 h with TGFβ1 (10 ng/mL) ± danegaptide (50–1000 nM). The LDH cytotoxicity assay kit II (Abcam) was used to quantify LDH according to the manufacturer’s instructions.

### 4.5. Crystal Violet Assay

This simple assay is used to measure the relative density of adhered cells to multi-well dishes. Crystal violet stains DNA and can be quantified colourimetrically after solubilisation. HK2 cells were seeded in 12-well plates and cultured in low-glucose DMEM/F12 (5 mM) for 48 h, prior to overnight serum starvation, and then subsequently stimulated for 48 h with TGFβ1 (10 ng/mL) ± danegaptide (50–1000 nM). The assay has been described previously [99]. Briefly, cells were fixed using paraformaldehyde for 10 min, washed with phosphate buffered saline (PBS) and incubated for 10 min at room temperature (RT) in a 0.1% crystal violet solution. After several more washes, the stain was solubilised using 1% SDS, and absorbance was measured by a plate reader.

### 4.6. Western Blotting

Preparation of cytosolic protein from HK2 cells and hPTECs, their separation by SDS-gel electrophoresis and transfer onto Immobilon-FL PVDF membranes have been described previously [12]. Membranes were blocked using Odyssey blocking buffer (LI-COR) and then probed overnight with antibodies against E-cadherin (1:1000), N-cadherin (1:1000), claudin-2 (1:500), ZO-1 (1:1000), collagen I (1:1000), collagen IV (1:2000), fibronectin (1:2000), laminin (1:500), ILK1 (1:500), β-catenin (1:2000), vimentin (1:500) and MMP3 (1:500). Bands were visualised using OdysseyFC and semi-quantified using ImageStudio (v5.2, LI-COR, Lincoln, NE, USA).

### 4.7. Transepithelial Resistance

HK2 cells were seeded (6 × 10^4^ cells/mL) onto Transwell filters (12 mm diameter, pore size 0.4 μM; Corning, NY, USA) and cultured in low-glucose DMEM/F12 (5 mM) for 48 h, serum-starved overnight and subsequently stimulated with TGFβ1 (10 ng/mL) ± danegaptide (100 nM) for 48 h. Transepithelial electrical resistance (TER) was measured using the EVOM apparatus (World Precision Instruments, Sarasota, FL, USA). A blank resistance measured from a well with medium alone was subtracted from each resistance reading.

### 4.8. Dye Uptake Studies

HK2 cells and hPTECs were seeded onto fluorodishes (22 mm diameter) and cultured in low-glucose DMEM/F12 (5 mmol/L) for 48 h. Following overnight serum starvation, the cells were incubated with TGFβ1 (10 ng/mL) ± danegaptide (50–100 nM) for 48 h. For subsequent steps, a balanced salt solution (BSS, pH 7.0) comprising of NaCl (137 mM), KCl (5.4 mM), MgSO_4_ (0.8 mM), Na_2_HPO_4_ (0.3 mM), KH_2_PO_4_ (0.4 mM), NaHCO_3_ (4.2 mM), HEPES (10 mM) and glucose (5 mM) was used. To induce dye uptake, cells were exposed to Ca^2+^-free BSS (zero CaCl_2_ + EGTA (1 mM)) plus carboxyfluorescein (200 µM) for 10 min, followed by a 5 min period in Ca^2+^-containing BSS (1.3 mM) plus carboxyfluorescein (200 µM). Dishes were subsequently washed with Ca^2+^-containing BSS (12 mL). Images were acquired with a Cool Snap HQ CCD camera (Roper Scientific, Gottingen, Germany) and Metamorph software (Universal Imaging Corp., Marlow, Bucks, UK). ImageJ was used to quantify dye uptake, where a region of interest (ROI) was drawn around each cell (approx. 10–15 cells/dish) and the mean pixel intensity measured. A background fluorescence value was subtracted from each ROI.

### 4.9. ATP Biosensing

ATP biosensors (Sarissa Biomedical, Coventry, UK) were used in a simultaneous dual-recording amperometric set-up, as described previously [12]. A null biosensor was used to account for non-specific electro-active artefacts and subtracted from the ATP trace. Glycerol (2 mM) was included in all recording solutions to enable ATP detection. HK2 cells were seeded on glass coverslips (10 mm diameter) in low-glucose DMEM/F12 (5 mmol/L) for 48 h prior to overnight serum starvation. The cells were then incubated with TGFβ1 (10 ng/mL) ± danegaptide (100 nM) for 48 h. The coverslips were transferred to a chamber containing Ca^2+^-containing BSS perfused at 6 mL/min (37 °C) and left for 10 min to acclimatise. ATP and null biosensors were bent and lowered so that the electrode lay parallel to the cellular monolayer. Once a stable baseline occurred, perfusion of Ca^2+^-free BSS stimulated hemichannel opening. After ATP release, Ca^2+^-containing BSS was used to close hemichannels, followed by a calibration solution of ATP (10 mM). Recordings were acquired at 4 Hz with a Micro CED (Mark2) interface using Spike (v8.03) software.

### 4.10. Inflammation Antibody Array

An inflammation antibody array (RnD Systems) assessed TGFβ1-induced regulation of 31 inflammatory markers. The array was performed by following the manufacturer’s instructions. Briefly, hPTECs were cultured in low-glucose DMEM/F12 (5 mM) for 48 h, prior to overnight serum starvation, and then subsequently stimulated for 48 h with TGFβ1 (10 ng/mL) ± danegaptide (100 nM). The cell lysates and supernatant were collected and incubated overnight with pre-blocked membranes spotted in duplicate with capture antibodies. An 800 CW fluorescent streptavidin/biotinylated cocktail mixture was used to visualise expression protein/antibody complexes by using Odyssey FC and semi-quantified using ImageStudio (v5.2, LI-COR).

### 4.11. Quantitative Real-Time PCR

RNA was extracted using an RNeasy mini kit (QIAGEN) and reverse-transcribed (Invitrogen). Real-time PCR (SYBR GreenER, Invitrogen) was performed using a StepOne Plus instrument (Applied Biosystems Inc, Foster City, CA, USA). cDNA expression was obtained by comparing to a standard curve of serially diluted cDNA. The following primers were used: p16 (forward: CTCGTGCTGATGCTACTGAGGA, reverse: GGTCGGCGCAGTTGGGCTCC), p21 (forward: AGGTGGACCTGGAGACTCTCAG, reverse: TCCTCTTGGAGAAGATCAGCCG), cyclin D1 (forward: TCTACACCGACAACTCCATCCG, reverse: TCTGGCATTTTGGAGAGGAAGTG), Klotho (forward: CCTCCTTTACCTGAAAATCAGCC, reverse: CAGGTCGGTAAACTGAGACAGAG) and GAPDH (forward: GTCTCCTCTGACTTCAACAGCG, reverse: ACCACCCTGTTGCTGTAGCCAA). Melt curve analysis confirmed primer specificity and checked for potential contamination.

### 4.12. Analysis

For all experiments, the low-glucose control (5 mM) was normalised to 100%, and all other conditions were compared to their respective controls. Statistical analysis was performed using a one-way ANOVA test with Tukey’s multiple-comparison post-test. Data are expressed as the mean ± SEM, with *n* denoting the sample number. A *p*-value of ≤0.05 signified statistical significance.

## Data Availability

The data presented in this study are available on request from the corresponding author. The data are not publicly available due to product development related to the research being reported, please see conflicts of interest statement for U.M.
